# Supplementary material for: Computational Study for the Unbinding Routes of β-N-Acetyl-d-Hexosaminidase Inhibitor: Insight from Steered Molecular Dynamics Simulations
Source: Int J Mol Sci. 2019 Mar 26;20(6):1516. doi: 10.3390/ijms20061516 (PMC6471479; doi:10.3390/ijms20061516)
Supplement: Supplementary file 1 [file ijms-20-01516-s001.pdf]

Computational study for the unbinding routes of β-N-Acetyl-D-hexosaminidase inhibitor: insight from steered molecular dynamics simulations

Song Hu, Xiao Zhao, Li Zhang*

Department of Applied Chemistry, College of Science, China Agricultural University, Beijing, China

Corresponding Authors* E-mail: zhang_li@cau.edu.cn

Contents:

Figure S1. (a) The binding pocket of OfHex1. (b) The structure of TMG-chitotriomycin.

Figure S2. The distance between O4 atom of the ligand and NE1 atom of the receptor versus time. CMD1 in black, CMD2 in red, CMD3 in blue.

Figure S3. Root-mean-square deviation (RMSD) variation of ligand with respect to simulation time. CMD1 in black, CMD2 in red, CMD3 in blue.

Figure S4. Root-mean-square deviation (RMSD) variation of receptor with respect to simulation time. CMD1 in black, CMD2 in red, CMD3 in blue.

Figure S5. Structure comparison of two loops for the conversional MDs (a) CMD1 (yellow), (b) CMD2 (pink) and (c) CMD3 (red) with the crystal structure (blue).

Figure S6. Dissociation routes of TMG-chitotriomycin from *Of*Hex1 identified clustered by CAVER from 100 snapshots of the last 5ns.

Figure S7. The average work versus the distance of ligand dissociation from the receptor

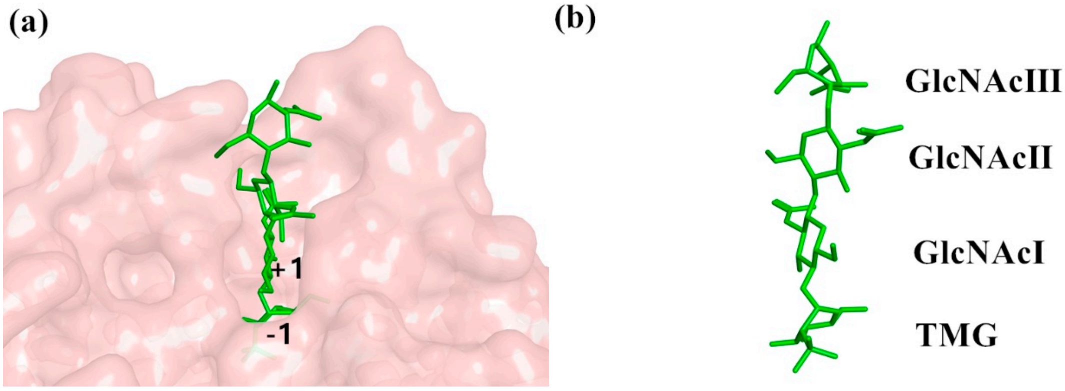

Figure S1. (a) The binding pocket of *Of*Hex1. (b) The structure of TMG-chitotriomycin.

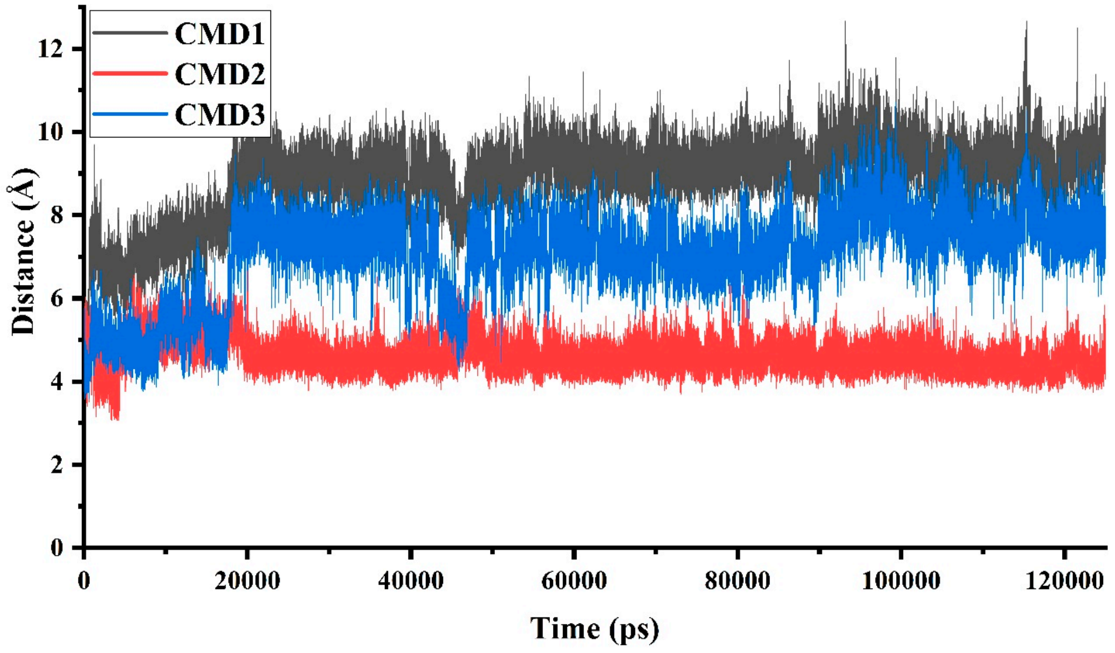

Figure S2. The distance between O4 atom of the ligand and NE1 atom of the receptor versus time. CMD1 in black, CMD2 in red, CMD3 in blue.

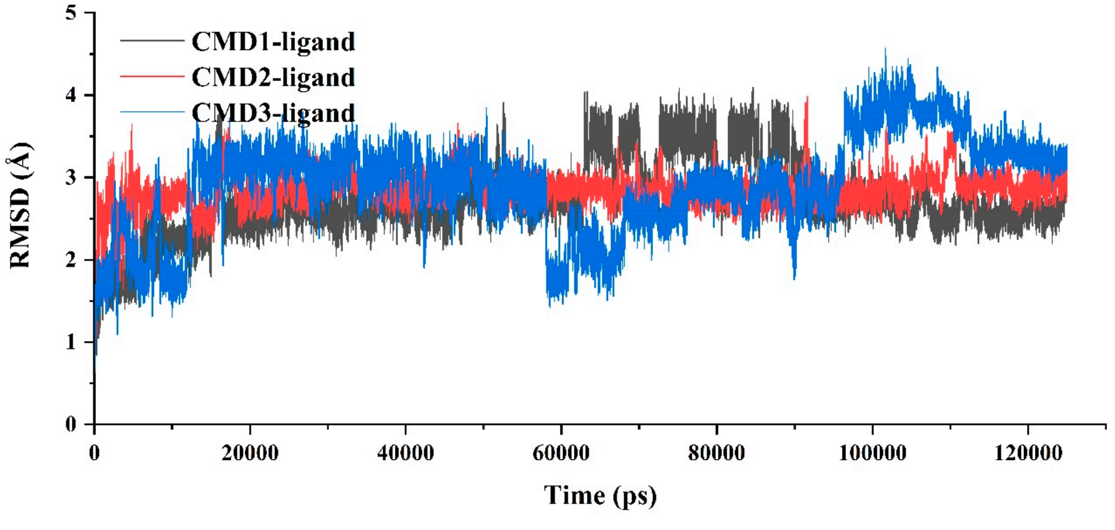

Figure S3. Root-mean-square deviation (RMSD) variation of ligand with respect to simulation time. CMD1 in black, CMD2 in red, CMD3 in blue.

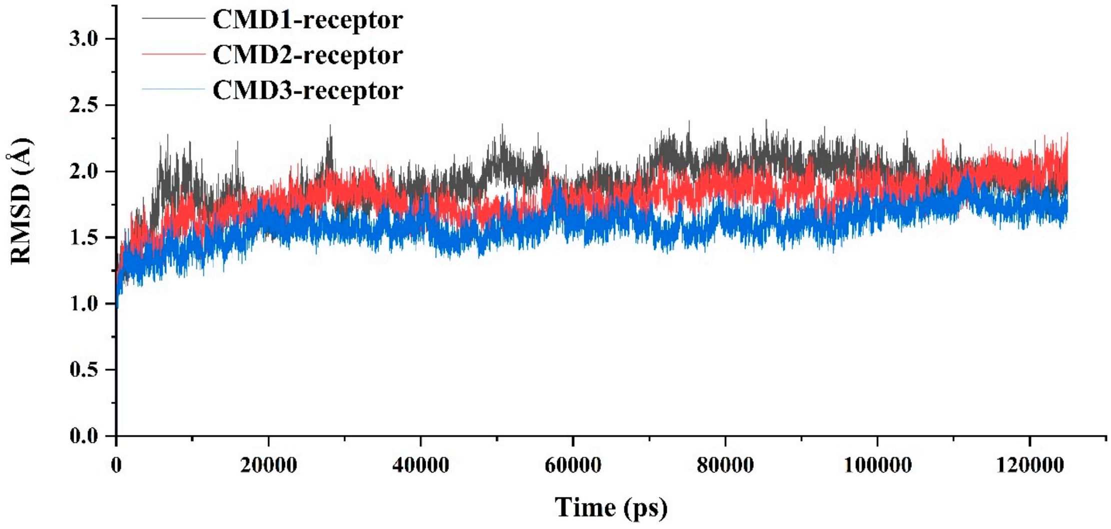

Figure S4. Root-mean-square deviation (RMSD) variation of receptor with respect to simulation time. CMD1 in black, CMD2 in red, CMD3 in blue.

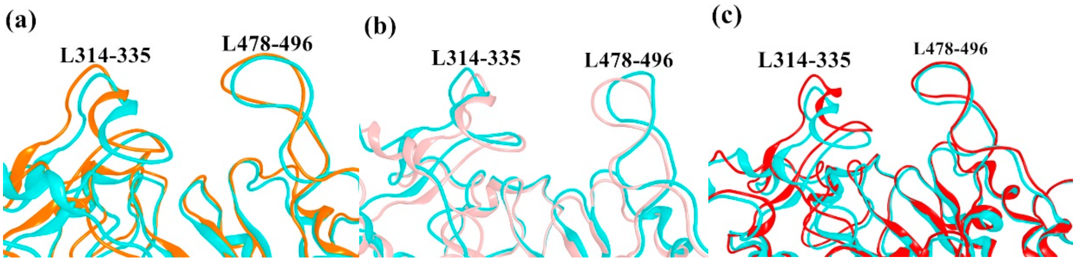

Figure S5. Structure comparison of two loops for the conversional MDs (a) CMD1 (yellow), (b) CMD2 (pink) and (c) CMD3 (red) with the crystal structure (blue).

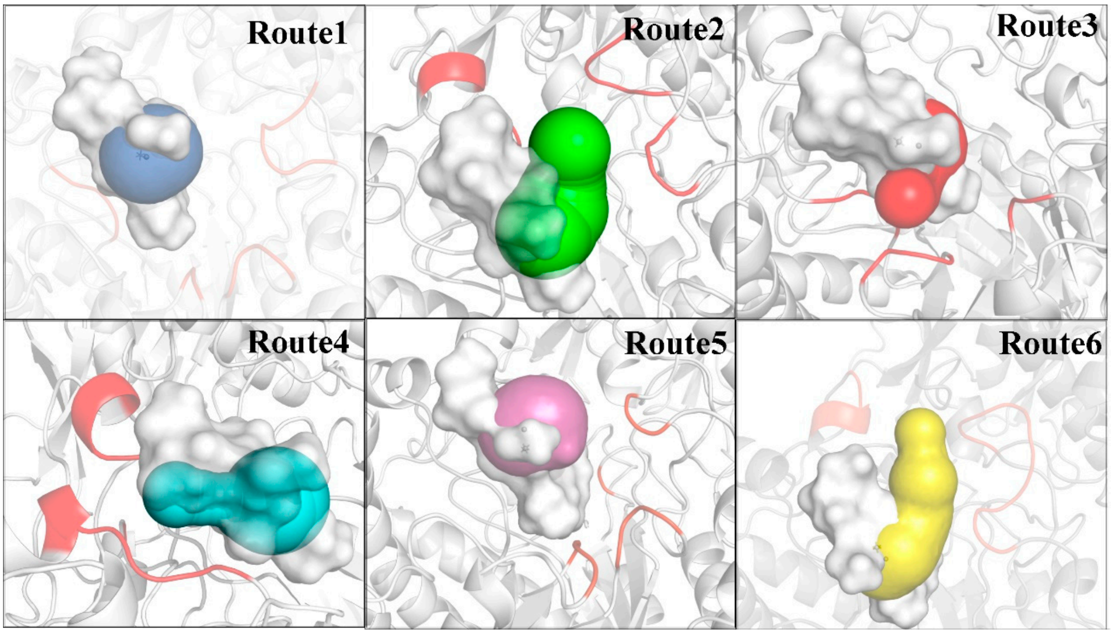

Figure S6. Dissociation routes of TMG-chitotriomycin from O*f*Hex1 clustered by CAVER from 100 snapshots of the last 5ns.

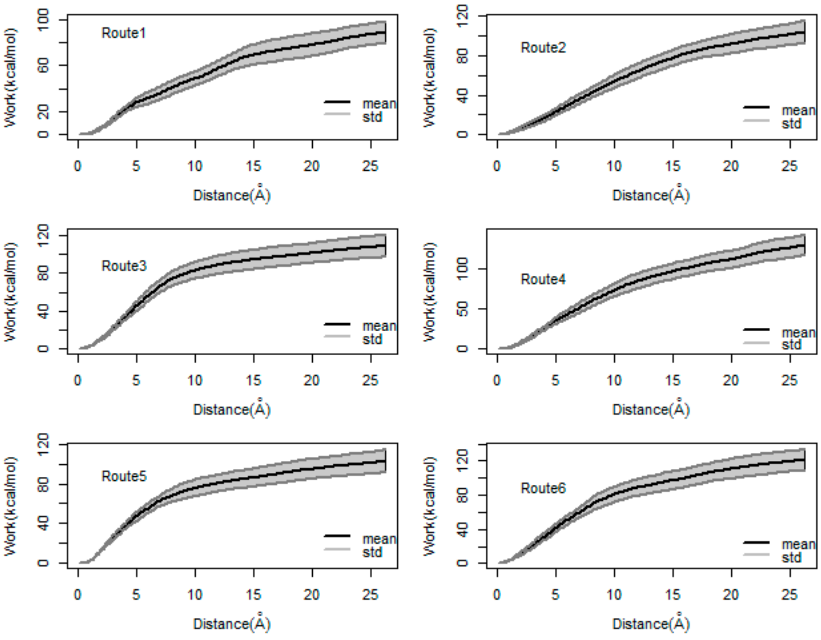

Figure S7. The average work versus the distance of ligand dissociation from the receptor
